# Characterization and Phylogenetic Analysis of the Complete Mitogenomes of *Valsa mali* and *Valsa pyri*

**DOI:** 10.3390/jof11050348

**Published:** 2025-04-30

**Authors:** Guoqing Xing, Shunpei Xie, Zhanxiang Qiao, Qingzhou Ma, Chao Xu, Yuehua Geng, Yashuang Guo, Rui Zang, Meng Zhang

**Affiliations:** College of Plant Protection, Henan Agricultural University, Zhengzhou 450002, China; xgq999@foxmail.com (G.X.); xieshunpei2020@163.com (S.X.); 18838020600@163.com (Z.Q.); 15237171177@163.com (Q.M.); chaoxu01@163.com (C.X.); gengyuehua@163.com (Y.G.); guoyashuang@henau.edu.cn (Y.G.)

**Keywords:** mitochondrial genome, collinearity, gene rearrangement, phylogenetic analysis, *Valsa mali*, *Valsa pyri*

## Abstract

Apple Valsa canker, caused by *Valsa mali* and *Valsa pyri*, is a devastating disease of apple trees and poses a severe threat to the sustainable development of apple production. Although the two species’ whole genomes have been sequenced, their mitochondrial genomes are still uncharacterized. In this study, the complete mitochondrial genomes of *V. mali* and *V. pyri* were assembled, annotated, and compared by bioinformatic methods. The results indicate that the mitogenomes are both circular DNA molecules with sizes of 213,406 bp and 128,022 bp, respectively. The AT skew values of the two *Valsa* species’ mitogenomes were positive, while the GC skew values were negative. Comparative mitogenome analysis revealed that the length and base composition of protein-coding genes (PCGs), rRNA genes, and tRNA genes differed between the two *Valsa* species. It was found that the expansion of *V. mali* was primarily attributable to the intronic regions. There are large numbers of interspersed repetitive sequences (IRS) in both *Valsa* mitogenomes; however, the proportion of IRS in *V. mali* (43.56%) was much higher than that in *V. pyri* (2.41%). The alignment of large fragments between the mitochondrial and nuclear genomes of both *V. mali* (1.73 kb) and *V. pyri* (5.17 kb) indicates that gene transfer between mitochondrial and nuclear genomes occurred during evolution. The ka/ks ratios for 15 core PCGs were below one, suggesting that these genes were subjected to purifying selection pressure. Comparative mitogenomics revealed that the two fungi had significant mitogenomic collinearity and large-scale gene rearrangements. The results of phylogenetic analysis based on Bayesian inference (BI) and maximum likelihood (ML) using a combined mitochondrial gene set confirmed that *V. mali* and *V. pyri* were fully independent taxa with a high bootstrap value of 100 (ML) and a high posterior probability of 1.0 (BI). This is the first report on the mitogenomes within the genus *Valsa*. These results will pave the way to understanding the evolution and differentiation of mitogenomes in the genus *Valsa*.

## 1. Introduction

Apple Valsa canker is one of the most economically important and destructive diseases in apple-producing areas of China [1,2,3], Japan [4,5], Korea [6], and the far eastern region of Russia [6]. The pathogen was initially identified as *Valsa mali* Miyabe et Yamada [7] and was later classified as a synonym of *V. certosperma* [8] Maire [9]. However, it was recently proven, based on the evidence from morphological characterization and molecular phylogenetic analysis, that *V. mali* is an evolutionarily independent species that is distinct from *V. certosperma* [3,10]. Furthermore, numerous cryptic divergences have been detected among *V. mali* isolates from apple (*Malus domestica* Borkh) and pear (*Pyrus communis*) [2]. Lu (1992) found that *V. mali* strains from pear were significantly different from those derived from apple on isozyme electrophoresis, although they have similar characteristics in culture [11]. Consequently, the *V. mali* strains from pear were classified as an independent variety and named *V. mali* var. *pyri* (*Vmp*), while the strains from apple were defined as *V. mali* var. *mali* (*Vmm*) for easier distinction [2,10].

In addition, an increasing amount of evidence supports the classification of the two varieties as independent species. They have been respectively reclassified as *V. mali* and *V. pyri* based on the combined sequences of rDNA-ITS, *β-tubulin*, and *EF1α* [2]. The genetic divergence in the rDNA-ITS sequence was lower, with a value of 1.4%, while in contrast, the divergences in the *β-tubulin* and *EF1α* sequences were significantly higher, with mean pairwise distances of 8.0% and 5.1%, respectively [2]. Furthermore, they exhibit distinct characteristics in culture on PDA plates and possess varying abilities to resist higher temperatures. The *V. pyri* colonies are almost always milky white, while *V. mali* colonies vary from white to light brown. The former grows normally on PDA plates at 37 °C, whereas the latter cannot grow at this temperature. More importantly, it is very interesting that *V. mali* strains are more aggressive on apple trees than on pear trees, while *V. pyri* exhibits higher virulence on pear trees than on apple trees. Finally, the whole-genome sequences revealed that the two species differ in size: the genome size of *V. mali* is 44.7 Mb, while the genome size of *V. pyri* is considerably smaller, at only 35.7 Mb [12]. The *V. mali* genome also contains a significantly greater number of repetitive elements, although the two species have nearly equal numbers of the genes associated with pathogenicity, secreted proteins, and proteases. The major difference between the two species lies in secondary metabolite gene clusters. Although many differences have been detected between the two species and phylogenetic analysis has been carried out based on multiple loci, including rDNA-ITS, *β-tubulin*, and *EF1α*, a more precise phylogenetic analysis at a broader scale, such as at the level of the mitogenome, has not yet been performed.

It is well known that the mitochondrion is an important double-membrane organelle in eukaryotic cells that is primarily responsible for producing adenosine triphosphate (ATP), the energy currency in living organisms [13]. Moreover, the mitochondrion is a semi-autonomous organelle in eukaryotic cells because mitochondria carry their own genetic material and possess the components for protein synthesis [14]. Beyond energy metabolism, the mitochondria play a very important role in other cell functions, such as Fe/S-cluster biosynthesis and amino acid metabolism, and they are also closely associated with apoptosis, cell senescence, virulence, and drug resistance [15,16,17]. Fungal mitogenomes usually contain 14 core protein-coding genes (PCGs), including three encoding ATP synthase subunits (*apt6*, *apt6*, and *atp9*), seven encoding subunits of reduced nicotinamide adenine dinucleotide ubiquinone oxidoreductase (*nad1*, *nad2*, *nad3*, *nad4*, *nad4L*, *nad5*, and *nad6*), three encoding subunits of cytochrome c oxidase (*cox1*, *cox2*, and *cox3*), and one encoding apocytochrome b oxidoreductase (*cob*) [14,17]. Moreover, the fungal mitogenome also encodes a ribosomal protein (*rps3*) and the RNA component for RNAse P (*rnpB* gene), along with two ribosomal RNA (rRNA) genes and 20~31 transfer RNA (tRNA) genes [18].

It is widely accepted that the mitogenome is a powerful tool for phylogenetic analysis in fungi due to its faster rate of evolution rate compared to the nuclear genome, as well as its lower recombination rate, conserved gene content, and uniparental inheritance; this finding has also been successfully leveraged in phylogenetic studies [19,20,21,22,23]. In addition, the relatively small size, circular-mapping topology, and multicopy nature of mitogenomes make their sequencing, assembly, and comparative analysis much easier than these are for the nuclear genome [17]. Recently, with the development of next-generation high-throughput sequencing technology, as well as the declining cost of sequencing, upgrades to related bioinformatics software, and algorithm optimization, more and more fungal mitogenomes have been sequenced, assembled, and annotated. According to the statistics, to date, a total of 3331 (accessed date: 28 March 2025) complete fungal mitogenome sequences have been submitted to the National Center for Biotechnology Information (NCBI) database (https://www.ncbi.nlm.nih.gov/genome/browse#!/organelles/). However, there is no available mitogenome information on *Valsa* species, and this has become a hindrance to fully understanding the important branch disease caused by the pathogenic *Valsa* fungi.

It has been reported that the size of the fungal mitogenome varies widely even within a genus. Kanzi et al. (2016) found that the mitogenome size of *Chrysoporthe deuterocubensis* was 124,412 bp and that of *C. austroafricana* was 190,834 bp, while that of *C. cubensis* was only 89,084 bp [24]. This variability can be attributed to differences in the length and number of introns, intergenic regions, repetitive DNA, open reading frames (ORFs) without defined functions, and homing endonuclease genes [19,25,26,27,28]. Among these factors, differences in the number and length of introns are considered to be the main ones contributing to the expansion or contraction of fungal mitochondrial genomes [17,29]. The introns in the fungal mitogenome can be divided into two groups, named group I and group II [13]. The introns in both groups are self-splicing DNA sequences [17]. The group I introns are the most numerous and can encode homing endonucleases [30,31]. In contrast, group II introns encode mostly reverse transcriptase-like ORFs [30]. However, to date, there have been no reports on mitochondrial introns in the genus *Valsa*.

Therefore, in this study, the mitogenomes of causal agents of apple tree *Valsa* canker, specifically *V. mali* and *V. pyri*, were assembled, annotated, and compared with the mitogenomes of other related pathogens. The principal aims of this study are as follows: (i) to reveal the mitogenome features of the two *Valsa* species; (ii) to investigate interspecific mitogenome variation between the two *Valsa* species; (iii) to clarify the phylogenetic position of the two *Valsa* species based on the datasets containing the mitogenome-encoded genes. This study represents the first report on mitogenomes of *Valsa* species. It will provide foundational reference sequences for further investigations of *Valsa* mitochondrial genomes and pave the way for a deeper understanding of the genetic evolution and population genetics of *Valsa*, as well as of species differentiation within the genus in the future.

## 2. Materials and Methods

### 2.1. Sample Collection and Fungal Pure Culture Obtain

Apple branches or twigs with typical canker symptoms were collected from a commercial orchard in Lin County, Henan Province, China. Pure cultures were obtained from the tissues located between the diseased tissues and healthy areas using the tissue-isolation methods described by Wang et al. (2011) and Zang et al. (2012) [10,32]. A total of 24 isolates were obtained. The isolates were identified at the species level based on morphological characteristics, sequence analysis of rDNA-ITS, and a PCR protocol utilizing species-specific primers developed by Zang et al. (2012) [32]. The obtained pure-culture isolates were stored at −80 °C in the ultra-low-temperature freezer (MECCAN DW-HL530, Hefei, China) at the Fungal Institute of Henan Agricultural University. The *V. pyri* strain was purchased from the Agricultural Culture Collection of China (ACCC36131).

### 2.2. Fungal DNA Extraction and Sequencing

Mycelium cakes were first obtained from the margins of fungal colonies that had been cultured at 25 °C for three days and then transferred onto the surface of potato dextrose medium (PDA) media, which was overlaid with a thin layer of sterile cellophane. The fungal total genomic DNA was extracted by the sodium dodecyl sulfate (SDS) method. The mycelia were harvested and ground into a powder with a sterile mortar under liquid nitrogen. The powder was suspended in 800 μL SDS extraction buffer, which contains 3% SDS, 50 mM EDTA, and 100 mM Tris-HCl (pH 8.0), transferred to a 2 mL-PCR tube, and then incubated at 65 °C for 1 h. Subsequently, an equal volume of phenol/chloroform/isoamyl alcohol (25:24:1) was added to the tubes, which were then gently inverted several times. The tubes were centrifuged at 4 °C and 12,000 rpm for 15 min. The supernatant (600 μL) was transferred to a new sterile 1.5 mL tube and eluted with an equal volume of chloroform. After centrifugation at 4 °C, 12,000 rpm for 10 min, the aqueous phase (350 μL) was collected and DNA was precipitated in isopropanol (250 μL). Finally, the mixed liquor was centrifuged at 4 °C and 12,000 rpm for 15 min, and the sedimented DNA could be seen at the bottom of the tubes. The supernatant was removed, and the DNA was washed twice with 75% ice-cold ethyl alcohol (500 μL) and then once with 100% ice-cold ethyl alcohol. A volume of 30 μL sterile deionized water was added dissolve the DNA after the ethyl alcohol had evaporated. The quality of the harvested DNA was qualitatively evaluated by agarose gel electrophoresis and quantified on the Qubit 2.0 fluorometer system (Thermo Scientific, Waltham, MA, USA). High-quality DNA was sent to the Novogene Company (Cambridge, UK), where the 350 bp sequencing library was constructed for short-read Illumina sequencing, and another 10K sequencing library was also constructed for long-read Nanopore sequencing.

### 2.3. Mitogenome Assembly and Annotation

The whole-genome sequencing was performed on the Illumina NovaSeq PE150 and Nanopore PromethION platforms (Illumina, San Diego, CA, USA). The adapters and low-quality short reads were filtered using Fastp v0.12.4 software [33]. The obtained cleaned paired-end data were used to assemble the complete mitogenome with NOVOPlasty v4.3.1 or Get_organelle v1.7.5 software. The *cox1*sequence of *Diaporthe longicolla* was used as the seed sequence in NOVOPlasty analysis. The parameters of Get_organelle were set as follows: k-mers 21,45,65,85,105, --max-rounds 15, -F fungus_mt.

If the assembly results could not be circularized using the two software programs, the Nanopore long-reads were assembled using Minimap2.1 and Racon 1.5.0. The mitochondrial-related contigs were identified by performing a Blastn search against the mitogenome sequence of *Diaporthe longicolla*, which served as the reference sequence. The software program bwa 0.7.17-r1188 was used to align the sequenced reads to the assembled mitogenome [34]. Samtools (v1.15.1) was used to convert the SAM file to a sorted BAM file.

The complete mitogenomes of both *V. mali* and *V. pyri* were annotated by combining the results of the MFannot website (https://megasun.bch.umontreal.ca/apps/mfannot/, accessed on 12 March 2024) annotation and MITOS WebServer (http://mitos2.bioinf.uni-leipzig.de/index.py, accessed on 12 March 2024) annotation using genetic code 4 (The Mold, Protozoan, and Coelenterate Mitochondrial Code and the Mycoplasma/Spiroplasma code). The annotation results of MFannot were checked by GeSeq [35] and then manually checked to confirm the gene boundaries [22]. The tRNA secondary structures were predicted by MITOS with default parameters and redrawn with Adobe Illustrator CS6. The graphical maps of the two *Valsa* mitogenomes were drawn using the Organellar Genome Draw Maps (OGDRAW) v.1.3.1 tool [36].

### 2.4. The Sequences Analysis

The base compositions of the two *Valsa* mitogenomes were analyzed using BioEdit 7.2.5. Strand asymmetry was assessed according to the following formulas: AT skew = [A − T]/[A+T], and GC skew = [G − C]/[G+C] [37]. MegaX and KaKs_Calculator2.0 were used to calculate the nonsynonymous substitution rates (Ka) and the synonymous substitution rates (Ks) for the 15 core PCGs in the two *Valsa* mitogenomes [38,39]. The Codon Usage module was used to analyze codon-usage bias in the Sequence Manipulation Suite (https://www.bioinformatics.org/sms2/codon_usage.html, accessed on 28 March 2024), based on genetic code 4 [40]. Intronic pairs were examined by EMBOSS Stretcher global alignment, and the intron–exon borders of PCGs were checked using exonerate 2.4.0 [41]. Genome syntenies of the two *Valsa* mitogenomes and representative species in other genera were analyzed using Mauve v2.4.0 [42].

### 2.5. The Repetitive Elements Analysis

The BLASTn searches of the whole mitogenomes against themselves were performed to detect whether there were interspersed repeats or intragenomic duplications of large fragments based on an E-value of 1 × 10^−10^. Simple repeat sequences (SSRs) were identified using the MISA-web microsatellite identification tools website (https://webblast.ipk-gatersleben.de/misa/, accessed on 9 April 2024). The tandem repeats in the two *Valsa* mitogenomes were searched using the Tandem Repeats Finder with the default parameters. Duplication events between the mitogenomes and nuclear genomes were detected using BLAST package 2.9.0+ [43].

### 2.6. Phylogenetic Tree Construction and Phylogenetic Analysis

To investigate the phylogenetic positions of *V. mali* and *V. pyri* in the order *Diaporthales*, a phylogenetic tree was constructed with 92 Ascomycetes species using the concatenated mitochondrial gene set, which included 14 core PCGs and the *rps3* gene. *Taphrina deforman* and *T. wiesneri* from Taphrinomycetes were selected as outgroups (Table S7). The single mitochondrial genes were selected using PhyloSuite v1.2.2 and aligned using the MAFFT plug-in in this software [44]. The aligned sequences were concatenated using the Concatenate Sequence module in this software to obtain a combined mitochondrial gene set. Partition-Finder 2.1.1 was applied to determine the best-fit models of evolution and the partitioning scheme for the gene set [45]. The phylogenetic tree was constructed using the maximum likelihood (ML) method and the Bayesian inference (BI) method. The BI analysis was carried out using MrBayes 3.2.6 software [46]. Two independent runs with four Markov chains each were conducted for 3,000,000 generations. Each run was sampled every 100 generations. The first 25% of samples were discarded as burn-in, and the remaining trees were used to calculate Bayesian posterior probabilities (BPP) in a 50% majority-rule consensus tree [47]. The software iqtree (v2.1.3) was used to construct the ML phylogenetic tree based on the combined gene set, and the bootstrap values (BS) were calculated using 1000 replicates to assess the node support [48].

## 3. Results

### 3.1. The Mitogenome Size and Organization Characteristics

**Figure 1:**
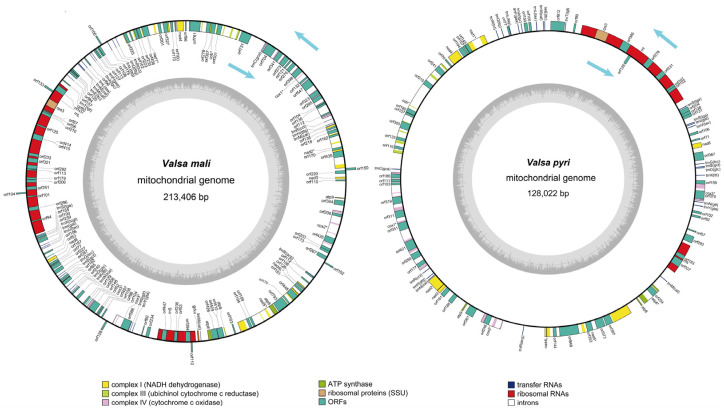
Circular maps of the mitogenomes of two *Valsa* species. Genes are represented by distinct colored blocks. Genes transcribed in a counterclockwise direction are located on the forward strand, while those transcribed in a clockwise direction are situated on the reverse strand. The inner ring illustrates the GC content.

A total of 5.49 G raw data from short reads and 7.92 G raw data from long reads were generated for *V. mali*. After processing, there were 5.55 G and 8.30 G of corresponding sequencing data for *V. pyri* and *V. mali*, respectively. The mitogenomes of the two *Valsa* species, *V. mali*, and *V. pyri*, were both assembled to circular DNA, with total sizes of 213,406 bp and 128,022 bp, respectively (Figure 1). It is clear that the *V. mali* mitogenome is significantly larger than that of *V. pyri*. The GC content of the *V. mali* mitogenome was 32.77%, while the value for *V. pyri* was 31.31%. These mitogenome GC contents were very similar. The AT skew values of two *Valsa* species mitogenomes was positive, while the GC skew value was negative. (Table S1). Whole sets of core PCGs were detected in the *V. mali* and *V. pyri* mitogenomes, including 15 typical core genes (*apt6*, *apt8*, *apt9*, *cob*, *cox1*, *cox2*, *cox3*, *nad1-6*, *nad4L*, and *rps3*). The *V. mali* mitogenome contains 35 introns distributed in the *nad1*, *nad2*, *nad5*, *cox1*, *cox2*, *cox3*, and *cob* genes, of which 33 belonged to group I and two belonged to group II. Similarly, The *V. pyri* mitogenome contained 22 introns distributed in the *atp6*, *nad1*, *nad5*, *cox1*, *cox2*, *cox3*, and *cob* genes, and all of them belong to group I. In addition, 54 and 22 free-standing ORFs (non-intron encoding ORFs) were respectively found in the *V. mali* and *V. pyri* mitogenomes. Meanwhile, 60 and 33 intronic ORFs encoding LAGLIDADG homing endonucleases, GIY-YIG homing endonucleases, and proteins with unknown functions were detected in the mitogenomes of *V. mali* and *V. pyri,* respectively. There was a slight difference in the numbers of ORFs encoding LAGLIDADG endonucleases and GIY-YIG homing endonucleases: 23 and 15, respectively, in the *V. pyri* mitogenome and 63 and 22, respectively, in the *V. mali* mitogenome. Notably, the *V. mali* mitogenome ORFs included almost three times as many ORFs encoding LAGLIDADG endonucleases as ORFs encoding GIY-YIG homing endonucleases (Table S2).

**Figure 2:**
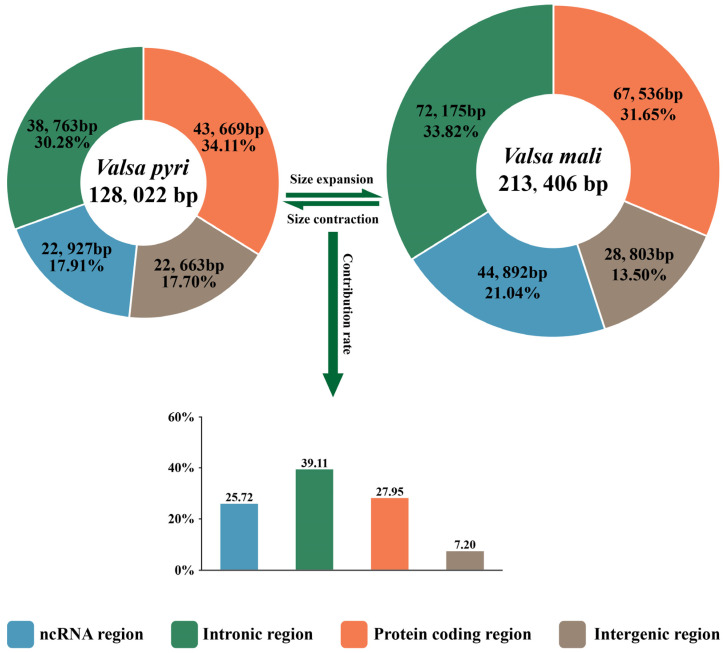
The ncRNA, intronic, protein-coding, and intergenic regions as proportions of the whole mitochondrial genomes of *V. pyri* and *V. mali*. The bottom histogram illustrates the contributions of different regions to the expansion and contraction of the mitochondrial genome in *V. mali*.

Protein-coding regions occupied the largest proportion of the *V. pyri* mitogenome, accounting for 34.11% of the mitogenome (Figure 2). The second-largest region was the intronic region, which accounting for 30.28%. The ncRNA (tRNA and rRNA) region and the intergenic region occupied almost the same proportions, respectively 17.91% and 17.7%. By contrast, in the mitogenome of *V. mali*, the largest region was the intronic region, which occupied 33.82% of the mitogenome. The second-largest region was the protein-coding region, which accounted for 31.65%, and the third-largest region was the ncRNA region, which accounted for 21.04% (Figure 2). Finally, the intergenic region took up the smallest proportion, 13.50%. The mitogenome of *V. mali* was 85,384 bp larger than that of *V. pyri*. The four regions played an important role in *V. mali* mitogenome expansion. The intronic region contributed the most to the *V. mali* mitogenome expansion, accounting for 39.11% of the difference. In addition, the ncRNA region, the protein-coding region, and the intergenic region also contributed to the expansion of *V. mali* mitogenome, respectively accounting for 25.72%, 27.95%, and 7.20% (Figure 2).

### 3.2. rRNA, tRNA, and Codon Usage Analysis

**Figure 3:**
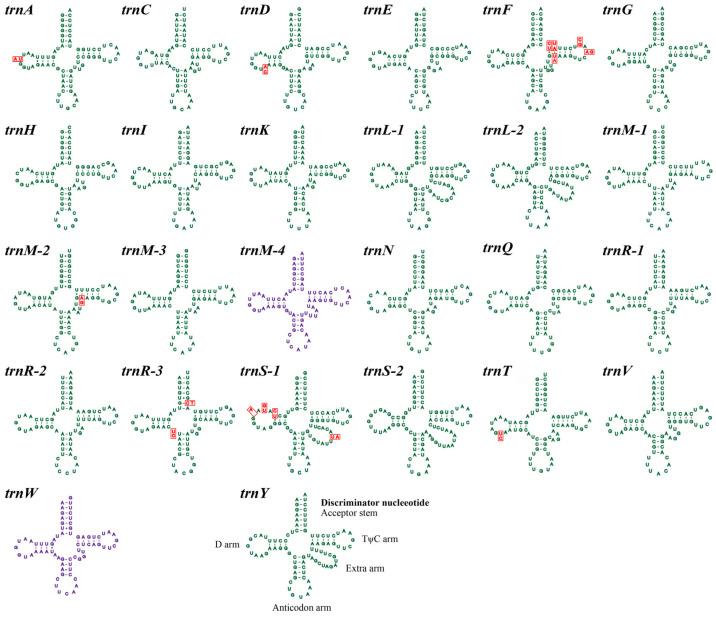
Putative secondary structures of tRNA genes identified in the mitogenomes of two *Valsa* species. The 24 tRNAs highlighted in green represent tRNAs shared by both *Valsa* species, while the tRNA in purple is unique to *V. mali*. Variable sites between the mitogenomes of *V. mali* and *V. pyri* are indicated in red.

The small subunit ribosomal RNA (*rns*) and large subunit ribosomal RNA (*rnl*) were both detected in both *Valsa* mitogenomes. One and six introns were respectively detected in the *rns* and *rnl* of the *V. pyri* mitogenome, whereas there is no intron was found in these genes in the *V. mali* mitogenome. In total, 26 and 24 tRNA genes were respectively detected in the mitogenomes of *V. mali* and *V. pyri*, with gene lengths ranging from 71 to 85 bp, mainly due to size variation in the extra arms. All tRNAs in the two *Valsa* mitogenomes can be folded into typical cloverleaf structures because no introns were found in these tRNA genes, which encode twenty kinds of standard amino acids (Figure 3). The two mitogenomes contained two tRNAs with different anticodons coding for serine and arginine and one tRNA with the same anticodon coding for methionine. Almost all tRNA genes were present in the two *Valsa* mitogenomes as single copies, except for the *trnL*, *trnM*, *trnR*, and *trnS* genes. *trnM* was present in four copies in *V. mali* mitogenome, while it was present in three copies in the *V. pyri* mitogenome. Additionally, three other tRNA genes (*trnL*, *trnR* and *trnS*) were observed to be present in multiple copies: the *trnR* gene was present in three copies, while *trnL* and *trnS* were present in two copies in each of the two mitogenomes. The *V. mali* mitogenome contained the *trnM-4* and *trnW* genes, whereas these two genes were absent from the *V. pyri* mitogenome.

**Figure 4:**
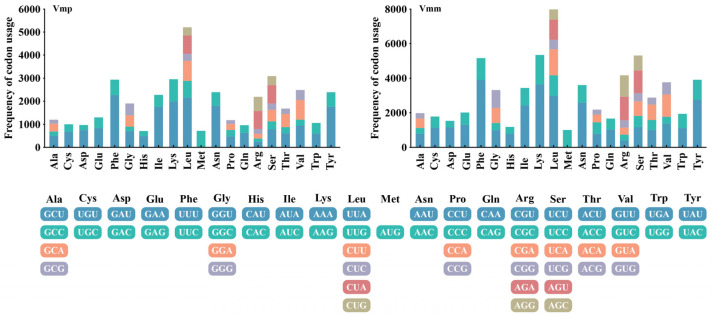
Codon usage in the mitogenomes of *V. mali* and *V. pyri*. The frequency of codon usage is plotted on the y-axis.

The results of codon-usage analysis showed that ATG was the most commonly used start codon in the core PCGs of the two *Valsa* mitogenomes. However, the *Cox1* gene of the two *Valsa* species used TTG as a start codon. The *nad4* gene of *V. mali* used TTG as the start codon, while that of *V. pyri* still used ATG as the start codon. The most frequently used stop codon was TAA, followed by TAG (Table S3). Meanwhile, the results also indicated that the most frequently used codons in the two *Valsa* mitogenomes were TTT (for phenylalanine; Phe), AAA (for lysine; Lys), TTA (for leucine; Leu), TAT (for tyrosine; Tyr), ATT (for isoleucine; Ile), and AAT (for asparagine; Asn). To some extent, the frequency of A and T used in codons leads to a relatively higher value of AT content in the two *Valsa* species (AT average: 67.96%) (Figure 4).

### 3.3. The Repeatitive Elements Analysis

A total of 21 and 1299 interspersed repetitive elements were respectively detected in the mitogenomes of *V. pyri* and *V. mali* through BLASTn searches of the two *Valsa* mitogenomes against themselves. The lengths of repeat sequences in the *V. pyri* mitogenome ranged from 52 to 418 bp, with pair-wise nucleotide similarities ranging from 79.11% to 96.15%. The largest repeat in *V. pyri* was located in the intergenic region between *nad5* and *atp8*. The repeat sequences accounted for 2.41% of the *V. pyri* mitogenome. The length of repeat sequences in the *V. mali* mitogenome ranged from 37 to 802 bp, with pair-wise nucleotide similarities ranging from 75.12% to 100%. The largest repeats in *V. mali* were observed in the intergenic region between *trnR* and *orf102*. Repeat sequences accounted for 43.56% of the *V. mali* mitogenome length. The repeat sequence of *V. mali* was much longer than that of *V. pyri* (Table S4).

A total of 19 and 33 tandem repeats were detected in the mitogenomes of *V. pyri* and *V. mali*, respectively. The longest tandem repeat sequence was found in *V. pyri* and extended over 99 bp while encompassing two repeat loci. Most tandem repeat sequences were duplicated once or thrice in the two *Valsa* mitogenomes, with the highest replication number (42) observed in the *V. mali* mitogenome. However, the longest repeat sequence was composed of 42 adenines (A), and this kind of tandem repeat sequence was absent from the *V. pyri* mitogenome. Tandem repeat sequences account for 0.72% and 0.10% of the mitogenome lengths of *V. pyri* and *V. mali* (Table S5).

To detect whether there were gene segments that had transferred between the nuclear and mitogenomes, the two *Valsa* mitogenomes were BLASTed against their nuclear genomes. Totals of 13 and 18 aligned fragments were respectively detected in the mitogenomes of *V. pyri* and *V. mali*. The lengths of these aligned segments ranged from 48 to 5172 bp, with sequence-identity values ranging from 76% to 100%. The largest fragment was located in the protein-coding regions of *cob* gene in the *V. pyri* mitogenome and had a length of 5172 bp. The largest aligned fragment in *V. mali* was detected between the fifth intron of the *nad5* gene and *orf103* and had a length of 1719 bp. The presence of large fragments that aligned between the mitochondrial and nuclear genomes indicates that genetic transfer between mitochondrial and nuclear genomes may have occurred during the evolution of *Valsa* species (Table S6).

### 3.4. Variation, Genetic Distance and Evolutionary Rates of 15 Core PCGs

**Figure 5:**
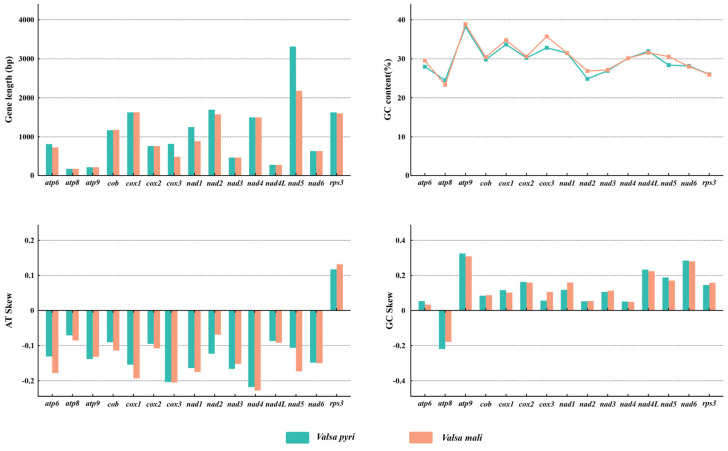
Variations in the lengths and base compositions of 15 protein-coding genes (PCGs) in the mitochondrial genomes of two *Valsa* species, including changes in PCG length, GC content, AT skew, and GC skew.

Among the 15 core PCGs detected in this study, eight genes including *nad1*, *nad2*, *nad4*, *nad5*, *cob*, *cox3*, *atp6*, and *rps3* were found to differ in length between the two *Valsa* species. Sequence alignment revealed that these variations predominantly occur at the gene initiation and termination regions. All of these genes except *cob* were obviously longer in *V. pyri* than *V. mali* (Figure 5). The GC contents of eight among the 15 core PCGs differed between the two *Valsa* species, while the other genes had almost the same GC contents. The *atp9* gene had the highest GC content, while *atp8* had the lowest GC content. The AT skew values of 14 core PCGs (all except *rps3*) were negative. Similarly, the GC skew values of 14 core PCGs (all except *atp8*) were positive (Figure 5).

**Figure 6:**
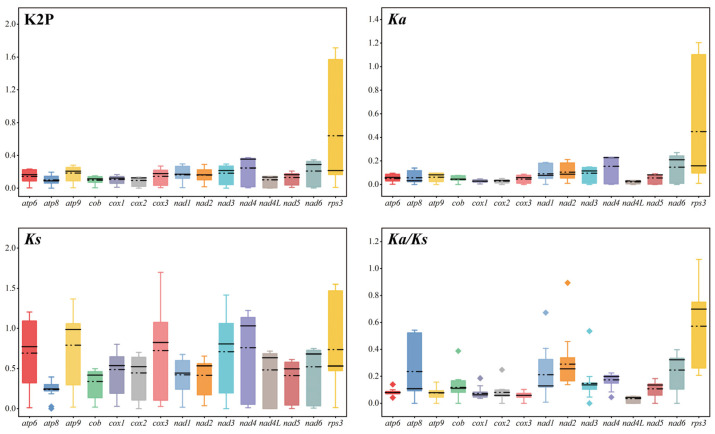
The genetic analysis of 15 protein-coding genes in six mitogenomes of *Diaporthales*. The black straight and dotted lines indicate the magnitudes of the median and mean values, respectively. K2P: the Kimura-2-parameter distance; Ka: the mean number of nonsynonymous substitutions per nonsynonymous site; Ks: the mean number of synonymous substitutions per synonymous site.

Among the 15 detected core PCGs, the *rps3* gene had the largest average value of Kimura-2-parameter (K2P) genetic distance, followed by *nad4*, *nad6*, and *nad3*, which demonstrates that these genes had differentiated greatly during the evolutionary process (Figure 6). The *atp8* gene showed the smallest K2P genetic distance among the six analyzed species (*Valsa mali*, *V. pyri*, *Chrysoporthe austroafricana*, *C. cubensis*, *C. deuterocubensis*, and *Diaporthe longicolla*) in the order *Diaporthales*, indicating that the gene is highly conserved. The *rps3* gene exhibited the largest non-synonymous substitution rate (*Ka*) among the analyzed PCGs, while *atp8* had the smallest *Ka* value. The synonymous substitution rate (*Ks*) of the *nad4* gene was the largest, while that of the *atp8* gene was the smallest among the analyzed species (Figure 6). Finally, the overall *Ka/Ks* values for all detected core PCGs were <1, indicating that these genes were subjected to purifying selection pressure.

### 3.5. Mitochondrial Gene Arrangement and Collinearity Analysis in Diaporthales

**Figure 7:**
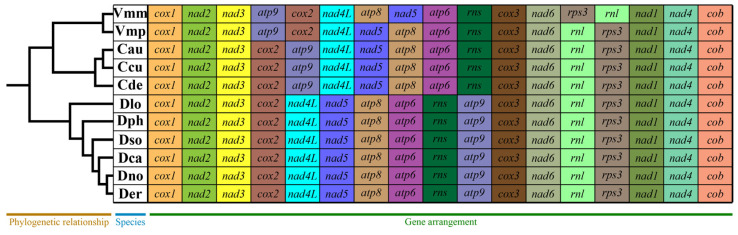
The mitochondrial gene-arrangement analyses of eleven species in the order *Diaporthales*. Genes are represented with different color blocks. Genes are shown in order of occurrence in the mitochondrial genome, starting from *cox1.* Fifteen core protein-coding genes and two rRNA genes were included in the gene-arrangement analysis. The phylogenetic positions of eleven species were determined using the Bayesian inference (BI) and maximum likelihood (ML) methods based on a concatenated mitochondrial gene sequence dataset.

The mitochondrial gene arrangement, including 15 core PCGs and two rRNA genes of eleven species (*Valsa mali*, *V. pyri*, *Chrysoporthe austroafricana*, *C. cubensis*, *C. deuterocubensis*, *Diaporthe longicolla*, *D. phaseolorum*, *D. sojae*, *D. caulivora*, *D. nobilis* and *D. eres*) in the order *Diaporthales* was analyzed. The results showed that the mitochondrial gene arrangement varied greatly in the order *Diaporthales*. Large-scale gene rearrangements, including gene relocations and position exchanges, were detected between species in different genera. However, the three examined species of *Chrysoporthe* and six species of *Diaporthe* each exhibited completely identical gene-arrangement patterns, suggesting these congeners may share closer phylogenetic relationships within their respective genera. By contrast, large-scale gene rearrangements were also detected between species in the same genus; the two *Valsa* species had different gene arrangements (Figure 7). This result indicated that the two *Valsa* species underwent different evolutionary processes.

**Figure 8:**
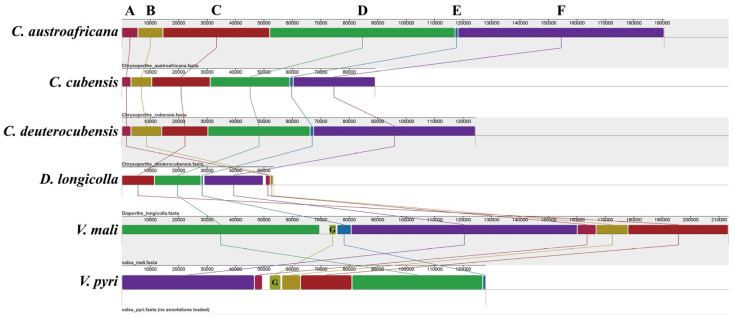
The gene collinearity analysis of six mitogenomes in *Diaporthales*. The blocks with the same colors represent homologous regions between different mitogenomes and are connected by the same color lines.

The mitogenome sizes of the six analyzed species (*Valsa mali*, *V. pyri*, *Chrysoporthe austroafricana*, *C. cubensis*, *C. deuterocubensis*, and *Diaporthe longicolla*) in *Diaporthales* varied greatly, ranging from 53,439 bp to 213,406 bp, with an average size of 133,199 bp. The mitogenome of *V. mali* was the largest among the analyzed *Diaporthales* mitogenomes, at almost four times that the size of the smallest mitogenome. In addition, the results of mitogenome collinearity analysis showed that the two *Valsa* mitogenomes can be divided into seven homologous regions. The relative positions of these homologous regions were highly variable between the two *Valsa* species (Figure 8). Homologous region G was absent from the genus *Chrysoporthe.* However, the relative positions of homologous regions were the same.

### 3.6. Phylogenetic Relationships Analysis

**Figure 9:**
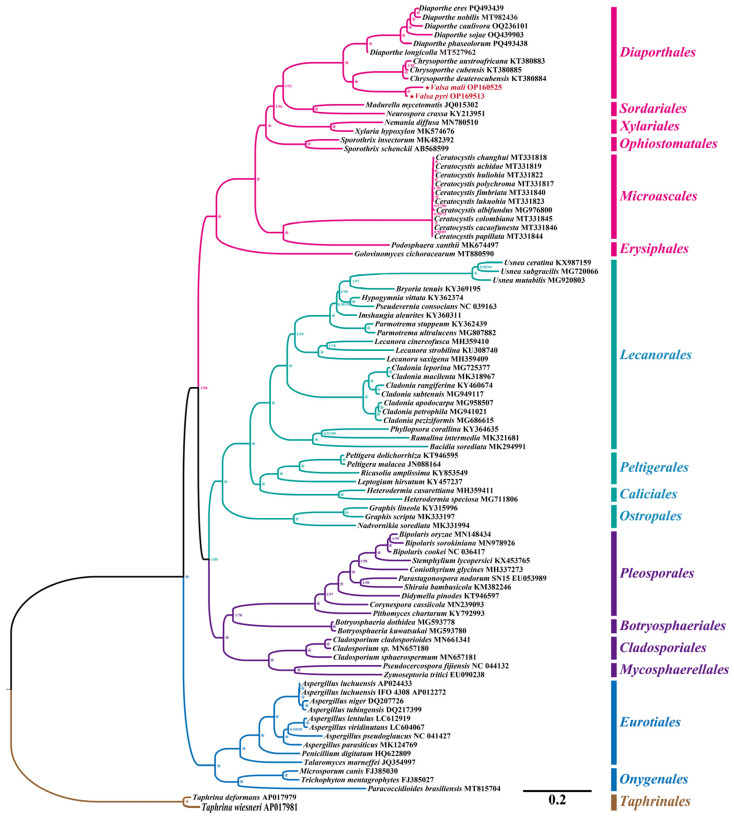
Molecular phylogeny of 92 Ascomycota species based on Bayesian inference (BI) and maximum likelihood (ML) analysis of 15 core protein-coding genes. Support values are Bayesian posterior probabilities (BPP) and bootstrap values (BS); these are placed before and after the slash, respectively. Asterisks indicate BPP and BS values of 1 and 100, respectively. Both Taphrina deforman and *T. wiesneri* from Taphrinomycetes were appointed as the outgroup.

The phylogenetic relationships analysis was carried out using the maximum likelihood (ML) method and a Bayesian inference (BI) method based on the concatenated mitogenome gene set including 14 core protein-coding genes and the *rps3* gene. It generated almost identical and well-supported tree topologies for 92 Ascomycota species (Figure 9). Almost all major branches in the ML tree and BI tree were well supported, with high bootstrap values. The results of the phylogenetic analysis showed that the 92 ascomycetous species can be divided into 17 major clades, corresponding to the orders of *Eurotiales*, *Onygenales*, *Pleosporales*, *Botryosphaeriales*, *Cladosporiales*, *Mycosphaerellales*, *Microascales*, *Diaporthales*, *Ophiostomatales*, *Xylariales*, *Sordariales*, *Lecanorales*, *Ostropales*, *Caliciales*, *Peltigerales*, *Erysiphales*, and *Taphrinales*, respectively. The six species within the order *Diaporthales* could be divided into three groups, wherein the first group was composed of one species in the genus *Diaporthe*, and the second group consisted of three species in the genus *Chrysoporthe* with a bootstrap value of 100 and a posterior probability value of 1.00. The two *Valsa* species are grouped together within the same cluster, with a bootstrap value of 100 and a poster probability value of 1.00. This topological structure shows that the two species have a very close evolutionary relationship; however, they are distinct sister species. This result also indicated that the phylogenetic analysis based on the mitogenome genes was a robust molecular marker by which to analyze the phylogenetic relationships of Ascomycota.

## 4. Discussion

In the study, the mitogenomes of two *Valsa* species, *V. pyri* and *V. mali*, were assembled and annotated respectively for the first time. The assembled mitogenomes of *V. pyri* and *V. mali* were both circularized DNA molecular with a size of 128,022 bp and 213,406 bp, respectively. The mitochondrial genome of *V. mali* is much bigger than that of *V. pyri*. It is well known that the size of the fungal mitochondrial genome varies greatly due to introns present within protein-coding genes, intergenic regions, repeat sequences, and plasmid-derived dynamic regions [13,23,26,27]. The *V. mali* mitogenome contains 35 introns, whereas the *V. pyri* mitogenome has only 22. In a comparison of the two mitogenomes, the intronic region contributed the most to the *V. mali* mitogenome expansion. Similar results were also observed in the mitogenomes of *Botryosphaeria dothidea*, *B. kuwatsukai* [21], *Exserohilum turcicum*, and *E. rostratum* [49]. In addition, the length of the repeat sequence of *V. mali* is much longer than that of *V. pyri*, which accounted for 43.56% of the *V. mali* mitogenome length, while the value in *V. pyri* is only 2.41%. The repeat sequences also played a very important role in the expansion of *V. mali* mitogenome. Although the proportion of intergenic regions differed little between the two *Valsa* mitogenomes, it also contributed to the expansion of the *V. mali* mitogenome.

The second parity rule states that each base in the complementary DNA strand has almost equal frequencies as long as there is no mutation or selection bias [50]. AT skews and GC skews were respectively detected in *V. mali* and *V. pyri.* The presence of AT skews and GC skews in different species demonstrates that mitogenomes of different species underwent different mutations or were subject to different environmental selection pressure. In addition, the *Ka/Ks* values for all core PCGs in the two *Valsa* species were <1, which revealed that they were subjected to stronger pressure of purifying selection [51].

The exchange of genetic material between the mitochondrial and nuclear genomes has been proven to be a common occurrence in various fungal species and promotes the differentiation of mitogenomes [37]. Meanwhile, the mitochondrial and nuclear genes work together synergistically to support the growth and development of fungi. It was commonly acknowledged that a majority of mitochondrial genes have been transferred to the nucleus, while some nuclear genes have also been discovered to have transferred to the mitogenome during the long process of evolution [52]. In the present study, it was discovered that more than ten larger fragments aligned, indicating likely transfer between mitochondrial genomes and the nuclear genome in the two *Valsa* species. This finding suggests that these species have experienced frequent natural gene transfers.

It has been reported that the mitochondrial gene arrangement can be used as an important clue to the phylogenetic relationships and evolutionary histories of eukaryotic species [37,53,54,55]. In the past two decades, with the development of high-throughput sequencing technology, more and more mitogenomes of eukaryotes have been sequenced., Mitochondrial gene rearrangement in animals has been widely studied, and many models associated with it have been established to reveal the mechanisms of mitogenome rearrangement [56]. Mitochondrial gene rearrangements are even more widespread and frequent in fungi than in animals [28,57]. Such gene rearrangements can be observed across the kingdom, with relevant species including but not limited to many mushroom-forming fungi, such as *Lyophyllum* spp. [47], *Pleurotus* spp. [58], *Cantharellus* spp. [59], *Russula* spp. [60]; plant pathogenic fungi, such as *Pseudocercospora fijiensis* [8], and *Bipolaris oryzae* [61]; and entomopathogenic fungi, such as *Beauveria caledonica* [62]. The gene rearrangements were mainly attritubed to nonhomologous, intrachromosomal recombination and the distribution of tRNA [57]. In the present study, significant mitochondrial gene rearrangement in the two *Valsa* species involved rRNA genes and core PCGs. The result showed that there are obvious differences between the two *Valsa* species. According to previous studies, fungal mitochondrial gene rearrangement may be attributed to the accumulation of repetitive sequences, especially in the intergenic regions [57]. In this study, a large number of repeat sequences was detected in the *V. mali* mitogenome, accounting for 43.56% of the length of the *V. mali* mitogenome. However, the proportion of repetitive sequences in *V. pyri* was much lower, only 2.41%. Compared with the other species in *Diaporthales*, gene rearrangements have been detected in the *V. pyri* mitogenome, which implies that other driving mechanisms may be responsible for fungal mitogenome rearrangement.

Most fungal species in the genus *Valsa* are important plant pathogens. However, no effective disease control strategy has been established despite many years of interest due to confusion with regard to species definition. It has been reported that identifying the *Valsa* species and their anamorphs *Cytospora* to the species level based solely on morphological characteristics is very difficult because of the overlapping and variable morphological characteristics of pathogenic anamorphs and the lack of teleomorphs [63]. Therefore, DNA sequences were introduced to study the phylogenetic relationships and species definitions. Up to now, rDNA internal transcribed spacer (rDNA-ITS), *β-tubulin* partial sequence and translation elongation factor (*EF1α*) have been widely used to explore the genetic relationships within the *Valsa* genus. It is still a difficult task to identify some species with more complex relationships using either single-gene or multiple-gene approaches to construct the phylogenetic trees. Here, a highly supported phylogenetic tree of 92 species in different orders was established based on the combined 15 mitochondrial core genes by using BI and ML phylogenetic analysis. The topological structures of the BI and ML trees indicated that all analyzed species were well clustered together or divided into different independent clades or subclades. The two *Valsa* species were clustered together with high values of 1.0 (BI tree) or 100 (ML tree) and were shown to be more closely related to the three *Chrysoporthe* species. This result showed that *V. mali* and *V. pyri* are two entirely different taxa. *V. mali* var. *mali,* and *V. mali* var. *pyri* should be renamed as *V. mali* and *V. pyri*, respectively. This result also demonstrates that the mitochondrial gene is an effective tool for the analysis of the phylogenetic relationship between *Valsa* and related genera. However, more mitogenomes of *Valsa* species must be obtained if we are to precisely understand the origins and evolutionary patterns of *Valsa*.

It was believed that the mitogenome was acquired from alphaproteobacteria by eukaryotic ancestors through endosymbiosis [64,65]. The main function of mitochondria is to serve as the suppliers of chemical energy in the form of ATP for aerobic respiration in eukaryotes [37]. However, recently, more and more studies have shown that mitochondria also play a crucial role in virulence because fungal growth, biofilm formation, and hyphal growth are regulated by the expression of mitochondrial genes [17]. It has been reported that the imperfect fission of mitochondria of *Pyricularia oryzae* affects conidiation, growth, and virulence, and that other changes in mitogenome genes affect the development of infection structures, invasion, and pathogenicity [66,67]. However, the corresponding relationships between the virulences of *Valsa* pathogens and their mitochondrial gene expression are still unknown. This problem should be studied in depth in the future to better understand their pathogenic mechanisms and to establish effective strategies to control plant disease.

## 5. Conclusions

In this study, the complete mitogenomes of *V. mali* and *V. pyri* were reported and compared with other mitogenomes from the order *Diaportliaccac*. The size of the *V. mali* mitogenome is 213,406 bp, while that of the *V. pyri* mitogenome is only 128,022 bp. *V. mali*’s mitogenome was significantly larger than that of *V. pyri*. The *V. mali* mitogenome included a set of 35 introns and 115 uORFs. By comparison, the *V. pyri* mitogenome contained 22 introns and 50 uORFs. In addition, there were significant differences in genome characteristics, such as gene length and base composition of PCGs, tRNAs, rRNAs, AT skew, and GC skew between *V. mali* and *V. pyri*. The intronic region was considered to be the main factor responsible for the difference in mitogenome size between the two *Valsa* genera. The two *Valsa* mitogenomes contain a large number of interspersed repetitive sequences (IRS); however, the proportion of IRS in *V. mali* (43.56%) was significantly greater than that observed in *V. pyri* (2.41%). It has also been found that large fragments may have transferred between the mitochondrial and nuclear genomes. The values of *Ka/Ks* for 15 core PCGs were <1, which indicated that these genes were subjected to purifying selection pressure. Significant mitogenomic collinearity and large-scale gene rearrangements were also found to have occurred in two *Valsa* mitogenomes. The use of a combined dataset of mitochondrial gene sequences for phylogenetic analysis resulted in well-supported phylogenetic trees using the Bayesian inference and the maximum likelihood methods. The topological structure indicated that *V. mali* and *V. pyri* were fully independent species. As this is the first report on mitogenomes in the genus *Valsa*, these results contribute to our understanding of the genetic evolution and species differentiation in the genus of *Valsa*.

## Data Availability

The original contributions presented in this study are included in the article and Supplementary Materials. Further inquiries can be directed to the corresponding authors.

## Supplementary Materials

The following supporting information can be downloaded at: https://www.mdpi.com/article/10.3390/jof11050348/s1, Table S1: Mitochondrial genomic data for two *Valsa* mitogenomes; Table S2: Characterization of the two *Valsa* mitogenomes; Table S3: Codon-usage analysis of two *Valsa* mitogenomes; Table S4: Local BLAST analysis of two *Valsa* species mitogenomes against themselves; Table S5: Tandem repeats detected in the mitogenomes of two *Valsa* species; Table S6: Results of local BLAST analysis of the mitogenomes of two *Valsa* species against the nuclear genome; Table S7: The species used for phylogenetic analysis in this study and their GenBank accession numbers.

## Abbreviations

The following abbreviations are used in this manuscript:

EF1α	Elongation factor-1 alpha
ITS	Internal transcribed space
TUB	beta-tubulin
BI	Bayesian inference
ML	Maximum likelihood
BPP	Bayesian posterior probability
K2P	Kimura-2-parameter
PCG	Protein-coding gene
rRNA	Ribosomal RNA
tRNA	Transfer RNA

## References

[1] Peng H.X., Wei X.Y., Xiao Y.X., Sun Y., Biggs A.R., Gleason M.L., Shang S.P., Zhu M.Q., Guo Y.Z., Sun G.Y. (2016). Management of Valsa Canker on Apple with Adjustments to Potassium Nutrition. Plant Dis..

[2] Wang X., Zang R., Yin Z., Kang Z., Huang L. (2014). Delimiting Cryptic Pathogen Species Causing Apple Valsa Canker with Multilocus Data. Ecol. Evol..

[3] Wang X., Shi C.M., Gleason M.L., Huang L. (2020). Fungal Species Associated with Apple Valsa Canker in East Asia. Phytopathol. Res..

[4] Abe K., Kotoda N., Kato H., Soejima J. (2007). Resistance Sources to Valsa Canker (*Valsa ceratosperma*) in a Germplasm Collection of Diverse *Malus* Species. Plant Breed..

[5] Togashi K. (1925). Some Studies on a Japanese Apple Canker and Its Causal Fungus, *Valsa mali*. J. Coll. Agric. Hokkaido Imp. Univ..

[6] Vasilyeva L., Kim W.G. (2000). *Valsa mali* Miyabe et Yamada, the Causal Fungus of Apple Tree Canker in East Asia. Mycobiology.

[7] Ideta A. (1909). Handbook of the Plant Diseases in Japan.

[8] Arcila-Galvis J.E., Arango R.E., Torres-Bonilla J.M., Arias T. (2021). The Mitochondrial Genome of a Plant Fungal Pathogen Pseudocercospora Fijiensis (Mycosphaerellaceae), Comparative Analysis and Diversification Times of the Sigatoka Disease Complex Using Fossil Calibrated Phylogenies. Life.

[9] Kobayashi T. (1970). Taxonomic Studies of Japanese Diaporthaceae with Special Reference to Their Life-Histories.

[10] Wang X., Wei J., Huang L., Kang Z. (2011). Re-Evaluation of Pathogens Causing Valsa Canker on Apple in China. Mycologia.

[11] Lu Y.J. (1992). Studies on the Pathogenic Fungus of Pear Canker Disease. Acta Phytopathol. Sin..

[12] Yin Z., Liu H., Li Z., Ke X., Dou D., Gao X., Song N., Dai Q., Wu Y., Xu J. (2015). Genome Sequence of *Valsa* Canker Pathogens Uncovers a Potential Adaptation of Colonization of Woody Bark. New Phytol..

[13] Sandor S., Zhang Y., Xu J. (2018). Fungal Mitochondrial Genomes and Genetic Polymorphisms. Appl. Microbiol. Biotechnol..

[14] Kouvelis V.N., Hausner G. (2022). Editorial: Mitochondrial Genomes and Mitochondrion Related Gene Insights to Fungal Evolution. Front. Microbiol..

[15] Calderone R., Li D., Traven A. (2015). System-Level Impact of Mitochondria on Fungal Virulence: To Metabolism and Beyond. FEMS Yeast Res..

[16] Chatre L., Ricchetti M. (2014). Are Mitochondria the Achilles’ Heel of the Kingdom Fungi?. Curr. Opin. Microbiol..

[17] Medina R., Franco M.E.E., Bartel L.C., Martinez Alcántara V., Saparrat M.C.N., Balatti P.A. (2020). Fungal Mitogenomes: Relevant Features to Planning Plant Disease Management. Front. Microbiol..

[18] Kulik T., Diepeningen A.D.V., Hausner G. (2021). Editorial: The Significance of Mitogenomics in Mycology. Fron. Microbiol..

[19] Fonseca P.L.C., De-Paula R.B., Araújo D.S., Tomé L.M.R., Mendes-Pereira T., Rodrigues W.F.C., Del-Bem L.E., Aguiar E.R.G.R., Góes-Neto A. (2021). Global Characterization of Fungal Mitogenomes: New Insights on Genomic Diversity and Dynamism of Coding Genes and Accessory Elements. Front. Microbiol..

[20] Song N., Geng Y., Li X. (2020). The Mitochondrial Genome of the Phytopathogenic Fungus Bipolaris Sorokiniana and the Utility of Mitochondrial Genome to Infer Phylogeny of Dothideomycetes. Front. Microbiol..

[21] Wang L., Zhang S., Li J., Zhang Y. (2018). Mitochondrial Genome, Comparative Analysis and Evolutionary Insights into the Entomopathogenic Fungus *Hirsutella thompsonii*. Environ. Microbiol..

[22] Wang B., Liang X., Hao X., Dang H., Hsiang T., Gleason M.L., Zhang R., Sun G. (2021). Comparison of Mitochondrial Genomes Provides Insights into Intron Dynamics and Evolution in *Botryosphaeria dothidea* and *B. kuwatsukai*. Environ. Microbiol..

[23] Zhang Y., Zhang S., Zhang G., Liu X., Wang C., Xu J. (2015). Comparison of Mitochondrial Genomes Provides Insights into Intron Dynamics and Evolution in the Caterpillar Fungus Cordyceps Militaris. Fungal Genet. Biol..

[24] Kanzi A.M., Wingfield B.D., Steenkamp E.T., Naidoo S., Van Der Merwe N.A. (2016). Intron Derived Size Polymorphism in the Mitochondrial Genomes of Closely Related *Chrysoporthe* Species. PLoS ONE.

[25] Araújo D.S., De-Paula R.B., Tomé L.M.R., Quintanilha-Peixoto G., Salvador-Montoya C.A., Del-Bem L.E., Badotti F., Azevedo V.A.C., Brenig B., Aguiar E.R.G.R. (2021). Comparative Mitogenomics of Agaricomycetes: Diversity, Abundance, Impact and Coding Potential of Putative Open-Reading Frames. Mitochondrion.

[26] Megarioti A.H., Kouvelis V.N. (2020). The Coevolution of Fungal Mitochondrial Introns and Their Homing Endonucleases (GIY-YIG and LAGLIDADG). Biol. Evol..

[27] Mukhopadhyay J., Hausner G. (2021). Organellar Introns in Fungi, Algae, and Plants. Cells.

[28] Zardoya R. (2020). Recent Advances in Understanding Mitochondrial Genome Diversity. F1000Research.

[29] Friedrich A., Jung P.P., Hou J., Neuvéglise C., Schacherer J. (2012). Comparative Mitochondrial Genomics within and among Yeast Species of the Lachancea Genus. PLoS ONE.

[30] Hafez M., Hausner G. (2012). Homing Endonucleases: DNA Scissors on a Mission. Genome.

[31] Li Y., Hu X.D., Yang R.H., Hsiang T., Wang K., Liang D.Q., Liang F., Cao D.M., Zhou F., Wen G. (2015). Complete Mitochondrial Genome of the Medicinal Fungus Ophiocordyceps Sinensis. Sci. Rep..

[32] Zang R., Yin Z., Ke X., Wang X., Li Z., Kang Z., Huang L. (2012). A Nested PCR Assay for Detecting *Valsa mali* Var. *Mali* in Different Tissues of Apple Trees. Plant Dis..

[33] Chen S., Zhou Y., Chen Y., Gu J. (2018). Fastp: An Ultra-Fast All-in-One FASTQ Preprocessor. Bioinformatics.

[34] Li H., Durbin R. (2009). Fast and Accurate Short Read Alignment with Burrows–Wheeler Transform. Bioinformatics.

[35] Tillich M., Lehwark P., Pellizzer T., Ulbricht-Jones E.S., Fischer A., Bock R., Greiner S. (2017). GeSeq–Versatile and Accurate Annotation of Organelle Genomes. Nucleic Acids Res..

[36] Greiner S., Lehwark P., Bock R. (2019). OrganellarGenomeDRAW (OGDRAW) Version 1.3.1: Expanded Toolkit for the Graphical Visualization of Organellar Genomes. Nucleic Acids Res..

[37] Li Q., Ren Y., Shi X., Peng L., Zhao J., Song Y., Zhao G. (2019). Comparative Mitochondrial Genome Analysis of Two Ectomycorrhizal Fungi (Rhizopogon) Reveals Dynamic Changes of Intron and Phylogenetic Relationships of the Subphylum Agaricomycotina. Int. J. Mol. Sci..

[38] Tamura K., Stecher G., Kumar S. (2021). MEGA11: Molecular Evolutionary Genetics Analysis Version 11. Mol. Biol. Evol..

[39] Wang D., Zhang Y., Zhang Z., Zhu J., Yu J. (2010). KaKs_Calculator 2.0: A Toolkit Incorporating Gamma-Series Methods and Sliding Window Strategies. Genom. Proteom. Bioinf..

[40] Stothard P. (2000). The Sequence Manipulation Suite: JavaScript Programs for Analyzing and Formatting Protein and DNA Sequences. BioTechniques.

[41] Slater G.S.C., Birney E. (2005). Automated Generation of Heuristics for Biological Sequence Comparison. BMC Bioinform..

[42] Darling A.C.E., Mau B., Blattner F.R., Perna N.T. (2004). Mauve: Multiple Alignment of Conserved Genomic Sequence With Rearrangements. Genome Res..

[43] Camacho C., Coulouris G., Avagyan V., Ma N., Papadopoulos J., Bealer K., Madden T.L. (2009). BLAST+: Architecture and Applications. BMC Bioinform..

[44] Zhang D., Gao F., Jakovlić I., Zou H., Zhang J., Li W.X., Wang G.T. (2020). PhyloSuite: An Integrated and Scalable Desktop Platform for Streamlined Molecular Sequence Data Management and Evolutionary Phylogenetics Studies. Mol. Ecol. Resour..

[45] Lanfear R., Frandsen P.B., Wright A.M., Senfeld T., Calcott B. (2016). PartitionFinder 2: New Methods for Selecting Partitioned Models of Evolution for Molecular and Morphological Phylogenetic Analyses. Mol. Biol. Evol..

[46] Ronquist F., Huelsenbeck J.P. (2003). MrBayes 3: Bayesian Phylogenetic Inference under Mixed Models. Bioinformatics.

[47] Li Q., Wang Q., Jin X., Chen Z., Xiong C., Li P., Zhao J., Huang W. (2019). Characterization and Comparison of the Mitochondrial Genomes from Two Lyophyllum Fungal Species and Insights into Phylogeny of Agaricomycetes. Int. J. Biol. Macromol..

[48] Trifinopoulos J., Nguyen L.T., von Haeseler A., Minh B.Q. (2016). W-IQ-TREE: A Fast Online Phylogenetic Tool for Maximum Likelihood Analysis. Nucleic Acids Res..

[49] Ma Q., Geng Y., Li Q., Cheng C., Zang R., Guo Y., Wu H., Xu C., Zhang M. (2022). Comparative Mitochondrial Genome Analyses Reveal Conserved Gene Arrangement but Massive Expansion/Contraction in Two Closely Related Exserohilum Pathogens. Comput. Struct. Biotec..

[50] Chen H., Sun S., Norenburg J.L., Sundberg P. (2014). Mutation and Selection Cause Codon Usage and Bias in Mitochondrial Genomes of Ribbon Worms (Nemertea). PLoS ONE.

[51] Yang Z., Nielsen R. (2000). Estimating Synonymous and Nonsynonymous Substitution Rates Under Realistic Evolutionary Models. Mol. Biol. Evol..

[52] Adams K.L., Palmer J.D. (2003). Evolution of Mitochondrial Gene Content: Gene Loss and Transfer to the Nucleus. Mol. Phylogenet. Evol..

[53] Beaudet D., Terrat Y., Halary S., De La Providencia I.E., Hijri M. (2013). Mitochondrial Genome Rearrangements in Glomus Species Triggered by Homologous Recombination between Distinct mtDNA Haplotypes. Genome Biol. Evol..

[54] Tang B.P., Xin Z.Z., Liu Y., Zhang D.Z., Wang Z.F., Zhang H.B., Chai X.Y., Zhou C.L., Liu Q.N. (2017). The Complete Mitochondrial Genome of Sesarmops Sinensis Reveals Gene Rearrangements and Phylogenetic Relationships in Brachyura. PLoS ONE.

[55] Zheng B.Y., Cao L.J., Tang P., Van Achterberg K., Hoffmann A.A., Chen H.Y., Chen X.X., Wei S.J. (2018). Gene Arrangement and Sequence of Mitochondrial Genomes Yield Insights into the Phylogeny and Evolution of Bees and Sphecid Wasps (Hymenoptera: Apoidea). Mol. Phylogenet. Evol..

[56] Boore J.L. (1999). Animal Mitochondrial Genomes. Nucleic Acids Res..

[57] Aguileta G., De Vienne D.M., Ross O.N., Hood M.E., Giraud T., Petit E., Gabaldón T. (2014). High Variability of Mitochondrial Gene Order among Fungi. Genome Biol. Evol..

[58] Li Q., Chen C., Xiong C., Jin X., Chen Z., Huang W. (2018). Comparative Mitogenomics Reveals Large-Scale Gene Rearrangements in the Mitochondrial Genome of Two Pleurotus Species. Appl. Microbiol. Biot..

[59] Li Q., Liao M., Yang M., Xiong C., Jin X., Chen Z., Huang W. (2018). Characterization of the Mitochondrial Genomes of Three Species in the Ectomycorrhizal Genus Cantharellus and Phylogeny of Agaricomycetes. Int. J. Biol. Macromol..

[60] Li Q., Wang Q., Chen C., Jin X., Chen Z., Xiong C., Li P., Zhao J., Huang W. (2018). Characterization and Comparative Mitogenomic Analysis of Six Newly Sequenced Mitochondrial Genomes from Ectomycorrhizal Fungi (Russula) and Phylogenetic Analysis of the Agaricomycetes. Int. J. Biol. Macromol..

[61] Song X., Geng Y., Xu C., Li J., Guo Y., Shi Y., Ma Q., Li Q., Zhang M. (2024). The Complete Mitochondrial Genomes of Five Critical Phytopathogenic *Bipolaris* Species: Features, Evolution, and Phylogeny. IMA Fungus.

[62] Glare T., Campbell M., Biggs P., Winter D., Durrant A., McKinnon A., Cox M. (2020). Mitochondrial Evolution in the Entomopathogenic Fungal Genus *Beauveria*. Arch. Insect Biochem. Physiol..

[63] Adams G.C., Roux J., Wingfield M.J. (2006). *Cytospora* Species (*Ascomycota*, *Diaporthales*, *Valsaceae*): Introduced and Native Pathogens of Trees in South Africa. Austral. Plant Pathol..

[64] Lang B.F., Gray M.W., Burger G. (1999). Mitochondrial Genome Evolution and the Origin of Eukaryotes. Annu. Rev. Genet..

[65] Muñoz-Gómez S.A., Wideman J.G., Roger A.J., Slamovits C.H. (2017). The Origin of Mitochondrial Cristae from Alphaproteobacteria. Mol. Biol. Evol..

[66] Patkar R.N., Ramos-Pamplona M., Gupta A.P., Fan Y., Naqvi N.I. (2012). Mitochondrial Β-oxidation Regulates Organellar Integrity and Is Necessary for Conidial Germination and Invasive Growth in *Magnaporthe oryzae*. Mol. Microbiol..

[67] Khan I.A., Ning G., Liu X., Feng X., Lin F., Lu J. (2015). Mitochondrial Fission Protein MoFis1 Mediates Conidiation and Is Required for Full Virulence of the Rice Blast Fungus Magnaporthe Oryzae. Microbiol. Res..

